# Tryptophan-PNA gc Conjugates Self-Assemble to Form Fibers

**DOI:** 10.1021/acs.bioconjchem.3c00200

**Published:** 2023-07-24

**Authors:** Andrea Mosseri, María Sancho-Albero, Flavia Anna Mercurio, Marilisa Leone, Luisa De Cola, Alessandra Romanelli

**Affiliations:** † Dipartimento di Scienze Farmaceutiche, Università Degli Studi di Milano, via Venezian 21, 20133 Milano, Italy; ‡ Department of Molecular Biochemistry and Pharmacology, Istituto di Ricerche Farmacologiche Mario Negri IRCCS, 20156 Milano, Italy; § Istituto di Biostrutture e Bioimmagini—CNR, via Pietro Castellino 111, 80131 Naples, Italy

## Abstract

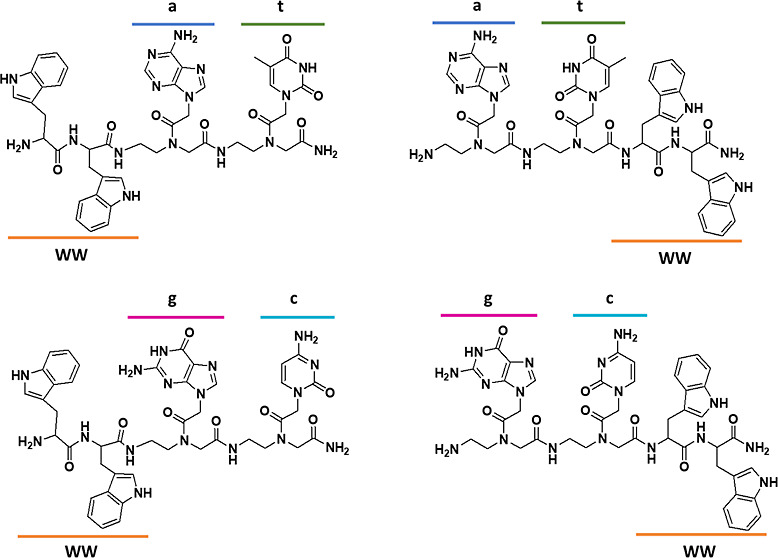

Peptide nucleic acids and their conjugates to peptides can self-assemble and generate complex architectures. In this work, we explored the self-assembly of PNA dimers conjugated to the dipeptide WW. Our studies suggest that the indole ring of tryptophan promotes aggregation of the conjugates. The onset of fluorescence is observed upon self-assembly. The structure of self-assembled WWgc is concentration-dependent, being spherical at low concentrations and fibrous at high concentrations. As suggested by molecular modeling studies, fibers are stabilized by stacking interactions between tryptophans and Watson-Crick hydrogen bonds between nucleobases.

## Introduction

Self-assembly of molecules containing both peptides and nucleobases such as nucleo-amino acids, peptide nucleic acids (PNA), or nucleosides conjugated to peptides has recently been investigated.^1−8^ These multi-component systems can generate a plethora of supramolecular structures, endowed with different chemical and physical properties.^9^ For example, they can arrange into fibers or spheres and can be used to produce hydrogels. A precise control over the macromolecular structure of such systems remains a challenge. In fact, the assembly is guided by low energy interactions, such as hydrogen bond or stacking between aromatic moieties, often competing between each other. In addition, the steric hindrance of assembling molecules plays a role in determining their arrangement. Interestingly, in self-assembled systems containing peptide and PNA, the presence of nucleobases triggers the onset of specific fluorescence signals, that can be exploited to monitor the assembly process.^3,10^ The biocompatibility and biodegradability of these molecules render them particularly interesting for biomedical applications.

In 2011, Xu and co-workers demonstrated that molecules composed of one nucleobase, one phenylalanine, and one d-glucosamine formed biocompatible and stable supramolecular hydrogels able to mediate the delivery of nucleic acids into the cytosol and nuclei of cells.^11^ In 2018, FF conjugated to a single A, T, or U nucleoside via a triazole linker, protected at the C or N terminus by a Boc group, was found to form microscopic nanospheres through aromatic stacking of the dipeptide.^12^ Conjugation of nucleobases or PNA to hydrogel forming peptides results in a significant improvement of the hydrogel physical properties. As an example, conjugates of nucleobases to the tripeptide FFY were proved to produce hydrogels with improved mechanical properties.^11,13^ Attachment of a single PNA unit to the hydrogel forming octapeptide FEFEFKFK resulted in modulation of the mechanical properties of the gels, and in particular in an increase in the hydrogel stiffness when guanine was used.^14^ Recently, it was demonstrated that mixing the PNA-peptide conjugates named p(tg)-FEFK and p(ac)-FEFK with complementary nucleobases produces a hydrogel endowed with stiffness and resistance to external stress highly improved as compared to hydrogel formed by the single components alone.^15^ Cryo-SEM experiments demonstrated that this gel is porous, contains homogeneous alveoli, and has great capability to entrap water molecules. Also, conjugates of Boc-FF to PNA protected on the nucleobases through a triazole linker assembled into hollow spheres, which were able to encapsulate doxorubicin and release it upon addition of a cationic dipeptide.^16^ We have reported self-assembly of PNA monomers and homodimers covalently linked to the peptide FF, and we observed formation of fibers, likely stabilized by hydrogen bonds between the peptide backbone forming beta sheet structures.^10^ When the PNA dimer “gc” was conjugated to FF, spheroidal structures were observed. Stacking interactions between nucleobases seem to play a role in the formation of such structures.^17^ The increase in the length of the peptide along with a change in the nucleobase composition drives formation of fibers: in fact, when FFFF is conjugated to the PNA dimer “at” the combination of antiparallel peptide sheets with Watson Crick hydrogen bonds between complementary bases results in formation of chiral fibers.^18^ PNAs bearing amino-acid side chains in the gamma position of the backbone were employed to produce amphiphile PNAs, termed bilingual PNAs. These PNAs with alanine or lysine side chains self-assemble, thanks to the interaction between the amphiphile side chain, and disassemble upon addition of oligonucleotides.^19^

The aim of this work is to explore the self-assembly of PNA dimers “at” and “gc” conjugated to the peptide ditryptophan WW. We wish to investigate how the chemical structure of the amino acid tryptophan (W) affects the aggregation properties and the structure of conjugates with PNAs. The tryptophan side chain, unlike that of phenylalanine, contains two condensed rings, a benzene and a pyrrole and possesses a permanent dipole. The indole ring of W is known to promote aggregation through aromatic stacking.^20^ In addition, the indole structure allows this amino acid to be involved also in electrostatic interactions through its π-conjugated electron cloud. Either π -cation interactions and π -anion interactions are possible; the first play a key role in the stabilization of protein structures. Finally, W may also be involved in hydrogen bonds through its NH group. In physiological conditions tryptophan self-assembles into cytotoxic nanofibers, characterized by amyloid mimicking and cross-seeding conformers.^21^ Co-assembly of D and L racemic mixtures of tryptophan yields rigid supramolecular materials with aromatic rings packed in a knob-to-hole fashion.^22^ It is reported that conjugation of the WW peptide to a DNA 12-mer results in the formation of aggregates, whose morphology is concentration-dependent.^23^ Recently, a molecule composed of l-tryptophan conjugated at its N-terminus with a thymine through a methylene carbonyl linker was found to be able to self-assemble and produce spherical aggregates, which can entrap curcumin.^24^ The presence of WW might change photophysical properties of PNA conjugates.

In this work, we have synthesized four WW conjugates, bearing the PNA “at” or “gc” at the N or C-terminal end of the peptide, and investigated the ability of these compounds to aggregate. Secondary structure and morphology studies were carried out on self-assembled WWat and WWgc. Finally, molecular modeling studies were performed to support the structural hypothesis that we formulated based on spectroscopic results. The large hydrophobic structure of the peptide WW combined to hydrogen bonds between guanine and cytosine triggers formation of fibers at high concentrations in WWgc conjugates.

## Results and Discussion

### Synthesis

**Figure 1:**
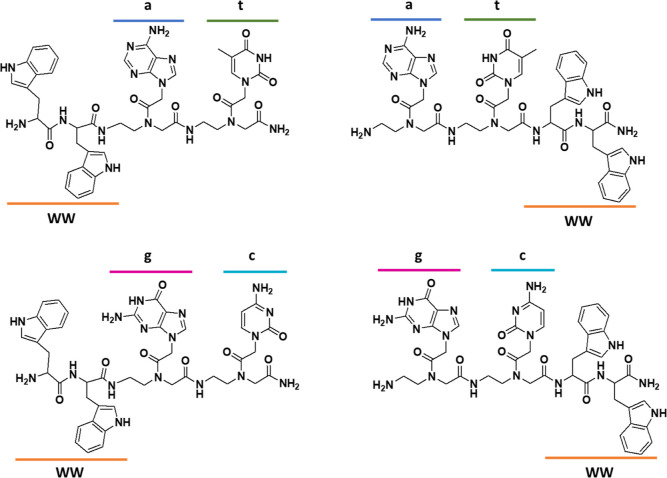
Chemical structure of the synthesized molecules. All l-amino-acids were employed.

The synthesis of the conjugates was performed by solid-phase techniques, as reported in the literature.^10,17^ Chemical structures of the conjugates are reported in Figure 1.

All conjugates were obtained as C-terminal amides. We synthesized conjugates with the PNA dimers “at” or “gc” at the C or the N-terminal end of the molecules. All molecules were purified by reverse phase-high-performance liquid chromatography (HPLC) and characterized by mass spectrometry (Supporting Information Figures S1 and S2). The PNA sequences were chosen in order to allow for the interaction between two different PNA-peptide units by base stacking and Watson-Crick hydrogen bonds. In fact, we have reported that stacking interactions stabilize aggregates when “gc” is conjugated to FF; formation of hydrogen bonds between “at” pairs has been reported in fibers formed by FFFF-at conjugates.^17,18^ In the last case, formation of an antiparallel beta sheet is driven by the interactions between the peptides; the nucleobases seem to play a role in “zipping” the sheets.

### Fluorescence Studies

The aggregation properties of the conjugates were assessed by fluorescence experiments. We exploited the emission properties of the anilino naphtalen sulfonic acid (ANS) probe to determine the minimal aggregation concentration of our compounds, following procedures reported in the literature for other peptide-PNA conjugates or nucleobases.^10^

**Figure 2:**
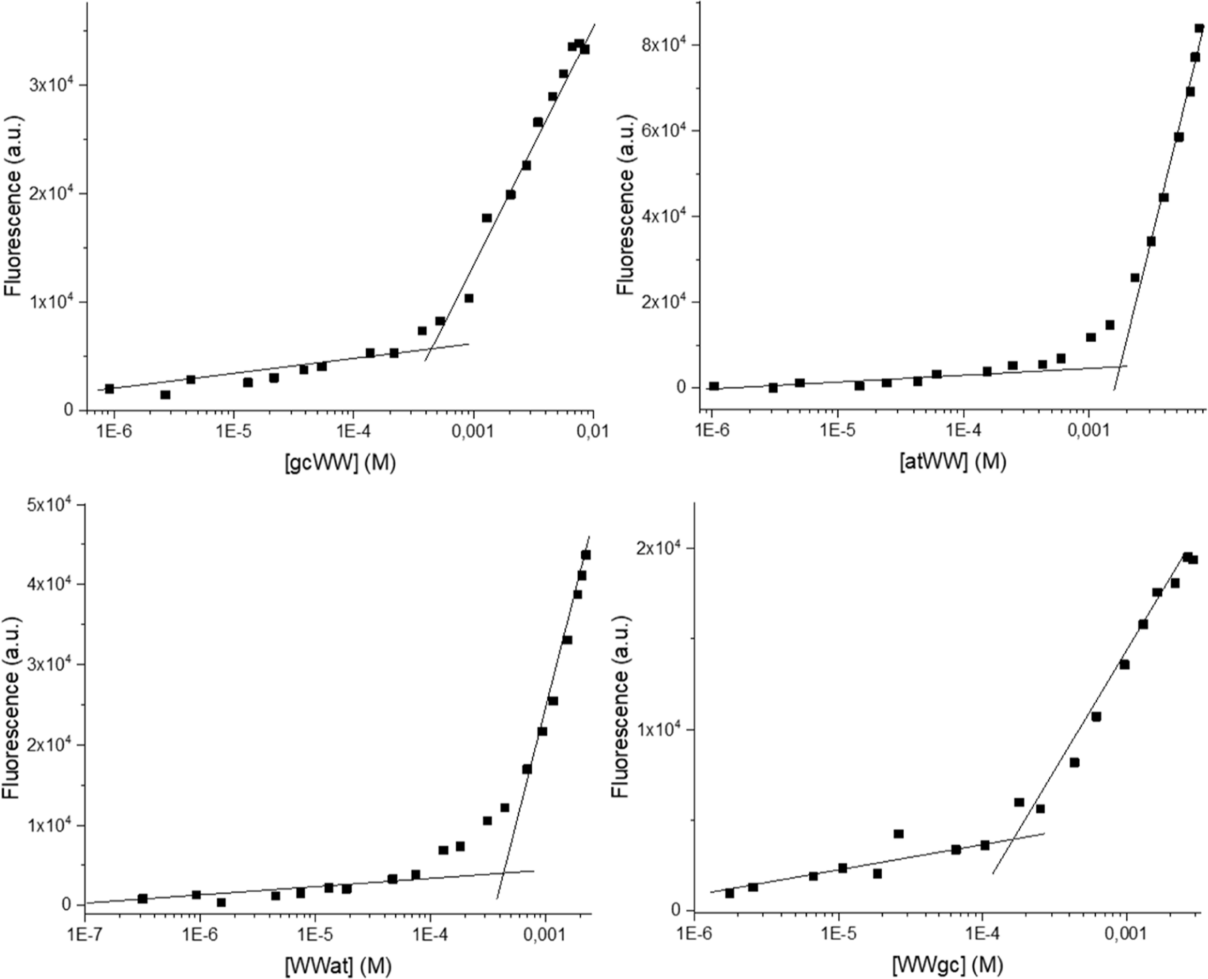
CAC determination: plots of the fluorescence intensity of the ANS fluorophore at λ = 490 nm versus peptide-PNA conjugate concentration.

The ANS solution was titrated with the PNA-peptide conjugates, and the fluorescence changes of ANS were monitored. The abrupt increase in the emission occurs when aggregation of the system begins. Plots of fluorescence emission vs compound concentrations are reported in Figure 2. The critical aggregation concentration (CAC) calculated for the conjugates with peptides at the C-terminus (atWW and gcWW) are higher as compared to the ones calculated for WWat and WWgc. We can speculate that the relative position of the PNA and peptide moieties affects the packing of the monomers. This trend is similar to that observed in gc PNA dimers conjugated to the dipeptide FF, where again lower CAC were observed when the peptide is at the N-terminal end of the molecules (0.17 mM for H-gcFF-NH_2_ vs 0.11 mM for H-FFgc-NH_2_).^17^ With the exception of atWW, CAC are in the low millimolar range (Table 1).

**Table 1:** CAC for the PNA-Peptide Conjugates

	WWat	WWgc	atWW	gcWW
CAC (M)	4.2 × 10^–4^	2.0 × 10^–4^	1.8 × 10^–3^	6.4 × 10^–4^

**Figure 3:**
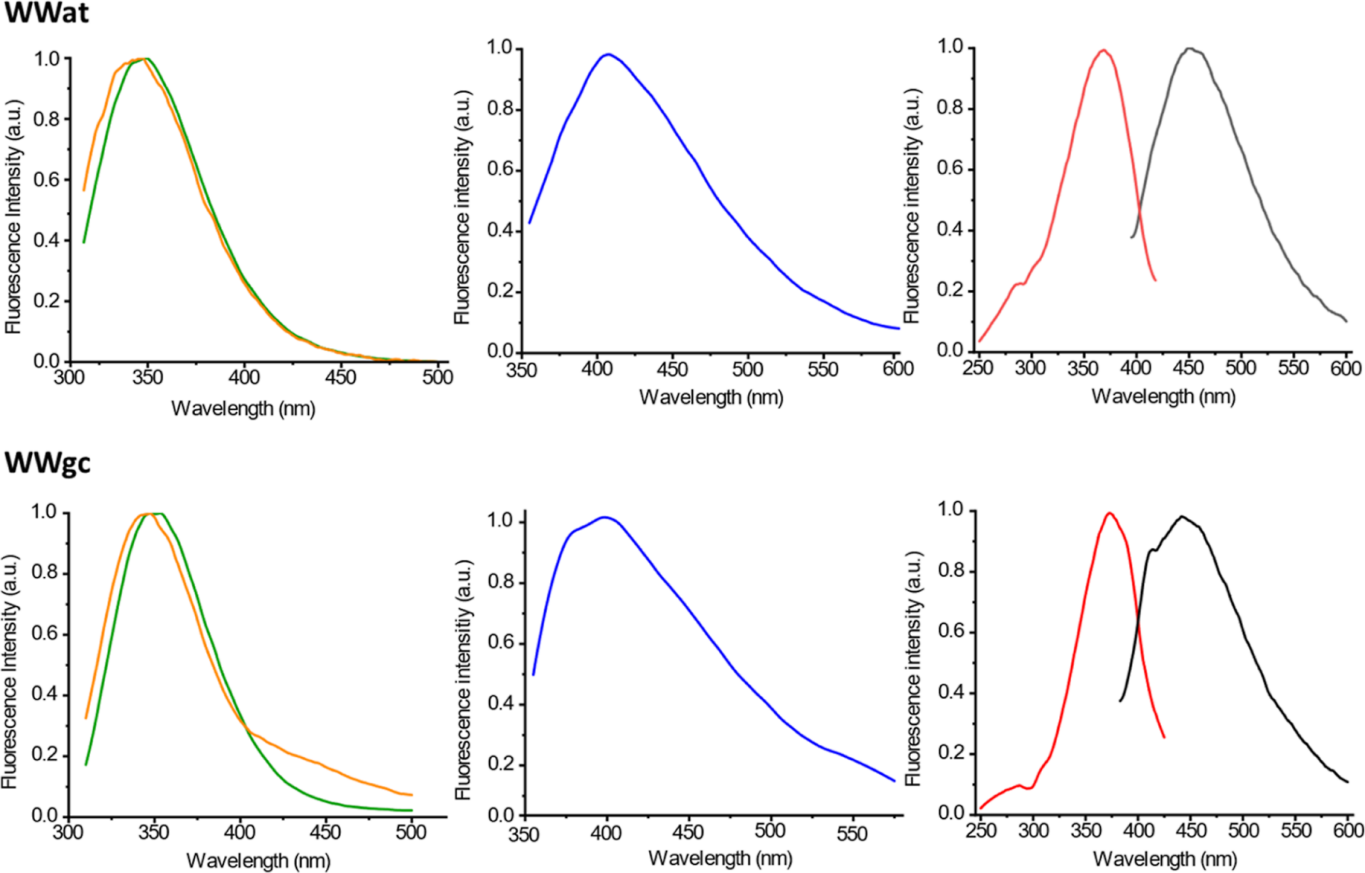
Fluorescence spectra of WWat and WWgc in water. From left to right: normalized emission spectrum λ_ex_: 280 nm (green line: 10 mg/mL; orange line: 0.5 mg/mL); emission spectrum λ_ex_: 330 nm (blue line) at 10 mg/mL; excitation (red line) and emission (black line) spectra with λ_ex_: 360 nm and λ_em_: 450 nm at 10 mg/mL.

Fluorescence properties of our conjugates were investigated in water at 10 mg/mL; we explored emission upon excitation at different wavelengths (Figure 3 and Supporting Information Figure S3). All compounds emit around 350 nm, due to the presence of the tryptophan. Inspection of the fluorescence spectra recorded at different concentrations for compounds containing the PNA dimer “gc” shows a red-shift in the emission maximum wavelength at increasing concentrations, that is significant (10 nm) for the WWgc (Supporting Information Figure S3). Fluorescence of tryptophan is very sensitive to its local environment, i.e., to polarity, hydrogen bonds or other non-covalent interactions that may occur.^25^ Usually tryptophan emission red shift is observed when the polarity of its environment increases, for example, when proteins denature and W residues move from a hydrophobic pocket to the solvent. In this case, the observed fluorescence red-shift may be related to a change in the organization of the aggregates at increasing concentrations, with tryptophan more exposed to the solvent at high concentrations.

Upon excitation at 330 nm, aggregates show emission around 400 nm. As reported for FFFFat and FFFFgc, we observe an intense band in the excitation spectrum that is not visible in the absorption spectrum and is reasonably due to excimers.^18^ This emission can be attributed to base aggregation; we have in fact previously demonstrated that at high concentration nucleobases do aggregate and emit fluorescence at this wavelength.^10^ In addition, similar signals are observed when PNA monomers and PNA homodimers are conjugated to various peptides, such as (FE)_2_(FK)_2_^14^ or FF.^17^ Finally, aggregates emit around 440–450 nm upon excitation at 360–370 nm (Figure 3 and Supporting Information Figure S3). Emission at longer wavelengths (440–450 nm) reasonably depends on the network of hydrogen bonds between the peptide backbone and is very similar to what previously observed in conjugates of “gc” or “at” to phenylalanine based peptides.^10,18^ The presence of the indole ring apparently does not affect emission properties of nucleobases in the aggregates.

### AFM Analysis

**Figure 4:**
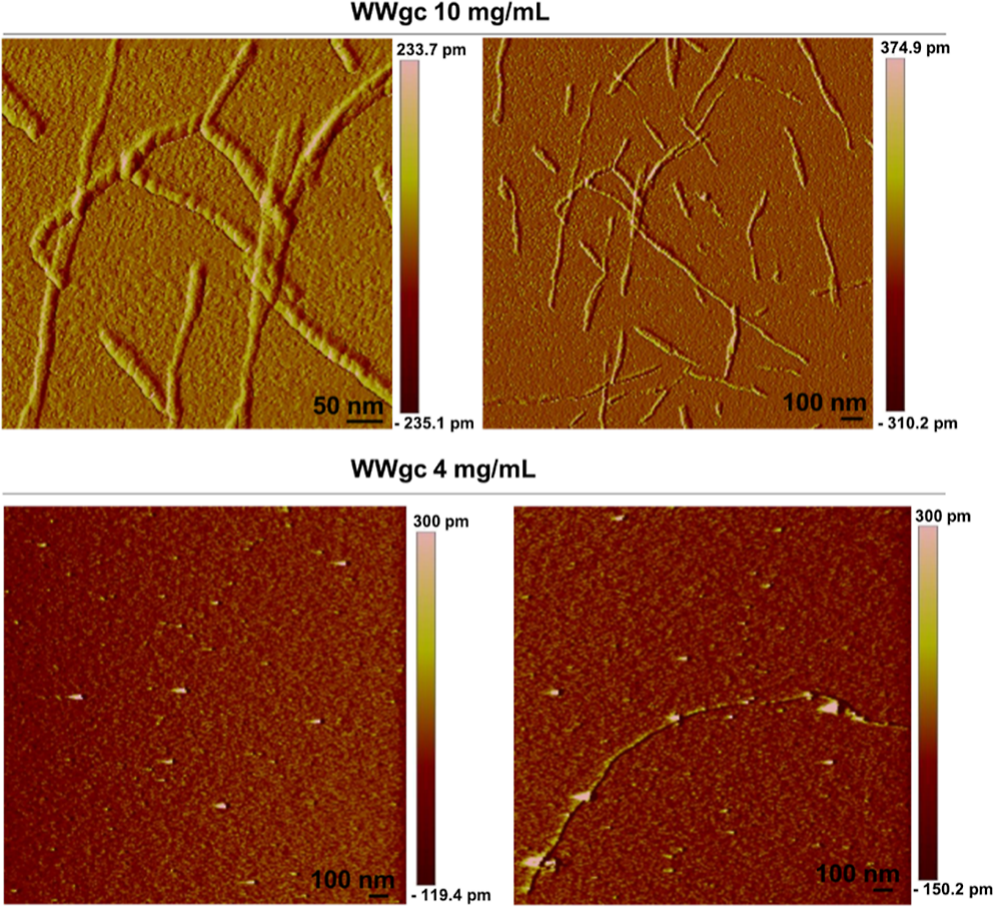
AFM analysis of WWgc comparing the fibers and the spherical aggregates obtained at 10 and 4 mg/mL, respectively. Both samples were dissolved in water.

In order to detect the morphology, the dimensions, and eventual chirality of the self-assembled aggregates, atomic force microscopy (AFM) imaging analysis was carried out. Figure S4 includes AFM images of atWW, WWat, gcWW, and WWgc at 10 mg/mL in H_2_O. According to these results, the initial analyses performed on samples containing “gc” and dissolved in water at 10 mg/mL revealed that only WWgc forms ordered fibers. These findings evidence how the position of the “gc” moiety in the molecule structure determines the self-assembling properties of the aggregates. Analyses were performed also at a lower concentration, i.e., 4 mg/mL. At this concentration, spherical particles are observed (Figure S5). Since in the literature it is reported that spherical structures obtained upon self-assembling of short peptide solutions evolve to nanofibrils,^26^ we monitored the morphology of self-assembled WWgc at a 4 mg/mL concentration after different incubation times. We did not observe formation of fibers after 17 days (see Supporting Information Figure S6). For this reason, we believe that formation of fibrils for WWgc is a concentration-dependent phenomenon. Figure 4 includes AFM images of WWgc comparing the fibers and the spherical aggregates obtained at 10 and 4 mg/mL, respectively.

To maintain the pH constant, the experiments were also performed in buffer at pH 5.5. As reported also in other cases, the pH of samples dissolved in water is not 7, but lower.^17^ Results obtained at pH 5.5 were comparable to those obtained in water, confirming that assembly occurs at a slightly acidic pH (Supporting Information Figures S5 and S7)

With the aim to promote aggregation reducing the polarity of the media, we analyzed the aggregates formed in buffer at pH 5.5 + 10% methanol (MeOH). Figure S7 shows AFM images of WWgc aggregates in buffer and in buffer with 10% MeOH at 10 mg/mL. When evaluating WWat compounds, AFM analyses revealed that in all buffers and at all tested concentrations only spherical objects were obtained (Supporting Information Figures S5 and S7). According to AFM results, WWat aggregates exhibited a round shape with a diameter around 50 nm when they were dissolved in water. In the case of the WWgc samples, even in the presence and in the absence of the methanol, fibers were observed.

In particular, analyses of the dimensions of the fibers obtained with the self-assembled WWgc sample revealed an average diameter of approximately 25 nm and a wide distribution in the length (200–900 nm) when the conjugates were dissolved at 10 mg/mL both in water or in buffer with 10% of MeOH (Figure S8).

The analysis of self-assembled structures carried out by AFM and the results obtained so far demonstrate that only WWgc self-assembles to give ordered structures exhibiting a fiber-like ordered structure. To address the question about chirality that is also visible in the fiber structures, we have performed circular dichroism (CD) experiments.

### CD Studies

**Figure 5:**
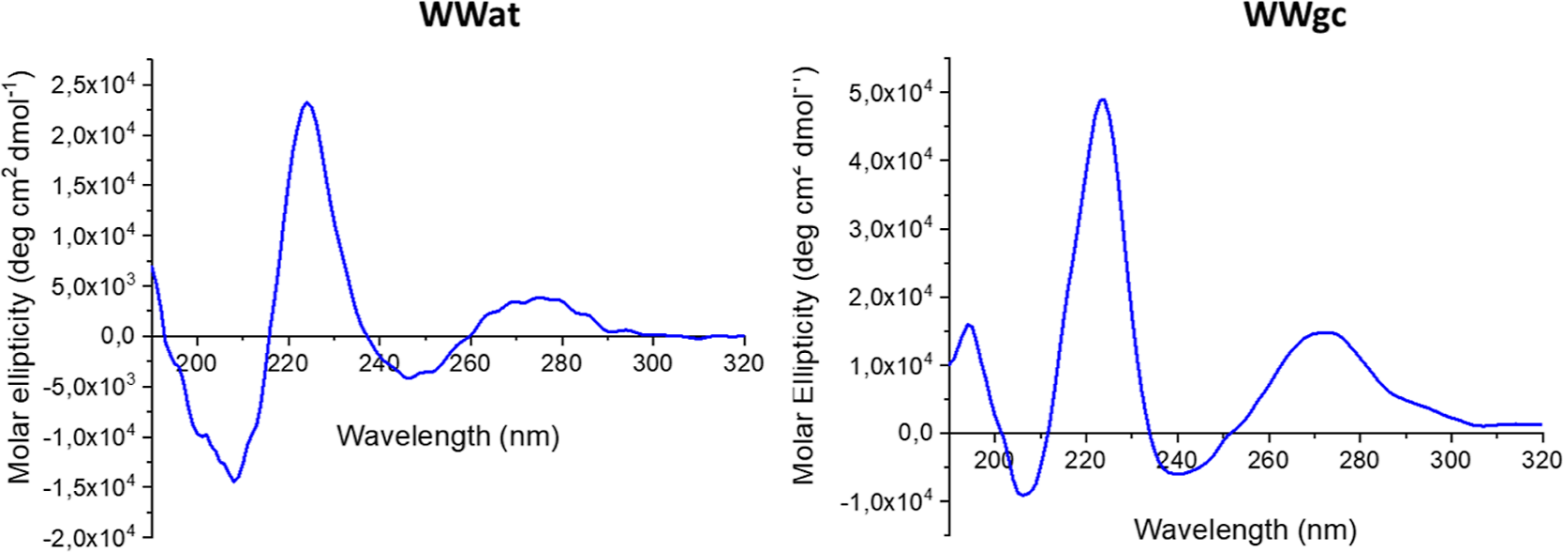
CD spectra of self-assembled WWgc and WWat in buffer at pH 5.5 at a concentration 10× CAC.

Secondary structure studies were performed by CD spectroscopy. WWgc and WWat spectra present a maximum around 270 nm, a minimum around 250 nm, and a maximum around 220 nm (Figure 5). Signals at higher wavelength can be attributed mainly to nucleobases stacking, while the signal at 220 nm depends on the presence and stacking of aromatic amino acids.^27^ Comparing the CD spectrum of B-DNA to spectra of WWgc and WWat between 250 and 300 nm, we observe a similarity in the position and sign of the signals.^28^ As B DNA is a right-handed helix, we could then speculate that WWgc fibers arrange as right-handed helices. The ratio between the intensity of signals at 220 and 240 nm as well as between signals at 260 and 240 nm is higher in the case of WWgc; this could be interpreted as due to a major contribution of bases’ and amino acids stacking in the stabilization of the structure.

### NMR Analyses

To gain further insights into structural features characterizing spherical aggregates and fibers, we performed solution NMR studies of WWgc samples (4 and 10 mg/mL concentrations) in water at pH 5. Comparison of one-dimensional (1D) [^1^H] NMR spectra of WWgc acquired at 10 mg/mL compound concentration at time zero (i.e., right after dissolving WWgc in water) and after 15 h of incubation at room temperature [i.e., after recording two-dimensional (2D) [^1^H, ^1^H] NMR spectra] shows a few changes possibly reflecting improved aggregation over time or formation of diverse aggregated species (Supporting Information Figure S9A). Indeed, after 15 h, the initial milky solution turned into a very viscous gel-like sample containing some precipitate as well. Interestingly, the spectrum of WWgc (10 mg/mL concentration) after 15 h is almost identical to that obtained for WWgc at 4 mg/mL concentration (Figure S9B). At the lower concentration (4 mg/mL), the WWgc solution appears clear, and even upon 15 h of incubation at room temperature, no changes in the spectra could be observed (Figure S9C), leading us to speculate that some stable, small, and soluble WWgc aggregates are present in solution alone. At 10 mg/mL under the experimental conditions employed for NMR studies, there are large aggregates and fibers that partially precipitate.

2D [^1^H, ^1^H] TOCSY spectra of WWgc samples at 4 and 10 mg/mL in the region close to 5–6 ppm (Supporting Information Figure S10) contain four cross-peaks arising from the correlation between H5 and H6 aromatic protons of cytosine.^18^ In isolated PNA dimers, limited rotation throughout the tertiary amide generates four rotamers, as the side chain carbonyl groups of the PNA can be faced either toward the N-terminus or the C-terminus. Investigation of NMR spectra points out that, similar to what was observed for the FFFFgc peptide/PNA conjugate, the gc PNA dimer acts as a spared fragment presenting multiple conformers (Figure S10).^18,29,30^ The NOESY spectrum of WWgc recorded at 10 mg/mL contains many negative NOEs that reflect a certain slower tumbling of the compound possibly induced by aggregation phenomena (Figure S11). We were unable to achieve unambiguous proton resonance assignments due to the presence in solution of multiple conformers (Figure S11A) and the extensive spectral overlaps particularly affecting the regions containing correlations from tryptophans and PNA bases aromatic protons (Figure S11B). Moreover, line broadening is also evident in the tryptophans aromatic protons correlation region (Figure S11B), leading us to speculate that they could play a pivotal role in formation of aggregates by providing intermolecular contacts. NMR data did not unequivocally point out gc base pairing as NOEs arising from amino and imino protons could not be identified likely due to the high solvent-exposure of these groups.^17,18^

As already described in our previous works on FFgc^17^ and FFFFgc,^18^ large molecular weight aggregates and protofibrils cannot be observed by solution NMR contrarily to disaggregated forms and/or small oligomers that can instead be detected.^31,32^ Thus, solution NMR data do not provide us with structural information on fibers that are likely insoluble and precipitate in the WWgc sample in water at 10 mg/mL. What we can observe by NMR are exclusively the smallest soluble WWgc aggregates that can be detected at both concentrations (4 and 10 mg/mL) and are likely characterized by tryptophan residues that, by interacting with each other through π–π intermolecular contacts, hide themselves from the solvent. According to this scenario, the more polar PNA dimers -gc- remain solvent exposed assuming multiple rotameric states. NMR data appear in line with fluorescence experiments, pointing out that tryptophans present larger solvent exposure in the fibers when WWgc concentrations are high while, at lower concentrations, tryptophans are immersed inside strictly packed spherical aggregates.

Computational molecular modeling was next employed to build speculative three-dimensional (3D) models of WWgc and WWat assemblies.

### 3D Models of WWgc Fibers

**Figure 6:**
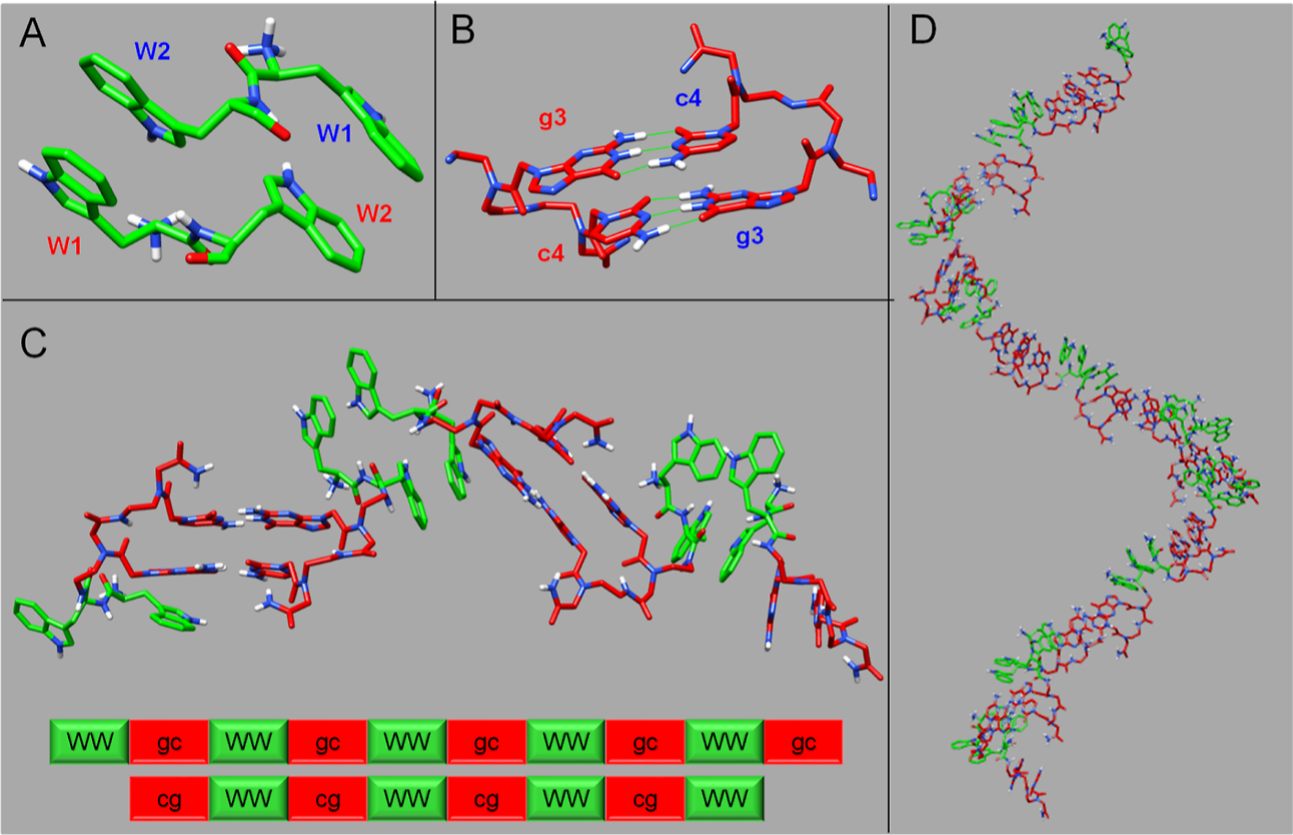
3D structural model of a WWgc helical bilayer. (A) Dimeric WW structural arrangement obtained by docking calculations. Tryptophan sequence numbers are indicated, and different colors are used for W belonging to different chains of the dimer. (B) gc Watson-Crick canonical base pairing in adjacent WWgc units. H-bonds are reported with green lines. (C) Assembly of a WWgc tetramer. The aggregates are stabilized by π–π aromatic interactions in between tryptophan side chains in two facing monomers, whose backbones run in an antiparallel manner, and by canonical Watson-Crick gc/cg base pairing. (D) Right-handed helical bilayer made up of 19 WWgc units. Fibers could be generated by side-by-side association of several bilayers. In all figures, tryptophan residues are colored in green and PNA bases in red.

In detail, to assemble a WWgc fiber, 3D coordinates of a WW dipeptide unit were generated with the software Chimera.^33^ The dipeptide was subsequently implemented as input receptor and ligand in Haddock^34^ to predict its dimeric form through molecular docking techniques. Haddock generated 200 structures and 190 of them were collected into 9 clusters, the most energetically favorable structure of the best cluster (Figure 6A) was next employed to develop WWgc conjugates.

In the dimeric WW structural organization, the tryptophan residues of the two monomeric units are placed in an anti-parallel orientation, and this configuration appears energetically favored as it is stabilized by intermolecular π–π stacking interactions in between side chains and the positive charges are positioned at the peptide N-termini far enough from each-others to avoid steric repulsions (Figure 6A).

CD data pointed out for WWgc formation of helical fibers with a right-handed structural organization. Thus, 3D atomic coordinates of gc/cg coupled base-pairs were extrapolated from the structure of a PNA duplex with a right-handed arrangement (pdb code 2KVJ,^35^Figure 6B). The PNA dimeric assemblies were joint at the C-termini of each WW dipeptide chain in the dimeric Haddock model (Figure 6C). After assembling several WW dimers and gc/cg duplexes, a bilayer with helical arrangement was observed. This model is characterized by π–π interactions in between tryptophan residues, flanked by gc/cg Watson-Crick base pairing involving PNA units (Figure 6D), and could constitute a basic building block mediating assembly of larger aggregates where different WWgc bilayers intertwine each other through aromatic inter-unit interactions mediated by tryptophans. The resulting structural arrangement could give rise to the fibers observed in AFM experiments (Figure 4).

### 3D Models of WWgc and WWat Spherical Particles

Spherical assemblies of WWgc and WWat were built assuming that different patterns of intermolecular contacts could be possible due to the peculiar chemical properties of these mixed peptide/PNA conjugates. In fact, WWgc and WWat have a certain amphiphilic character due to the hydrophobic stretch represented by the WW dipeptide aromatic side chains and a certain hydrophilicity mainly provided by the PNA dimers. Nevertheless, apart from the aromatic character, the tryptophan residue possesses also a polar indole ring whose proton can be involved into H-bonding and mediate self-assembly. Indeed, it has been described in literature formation of vesicular structures made up by *N*-alkylindoles^36^ and conversion of biotin fibers to spherical structures after conjugation to a WW di-peptide.^37^ In a recent study on nanomaterials generated by F or W self-assembly, the crucial role of H-bonding through the W indolic H_N_ was further stressed out.^22^

**Figure 7:**
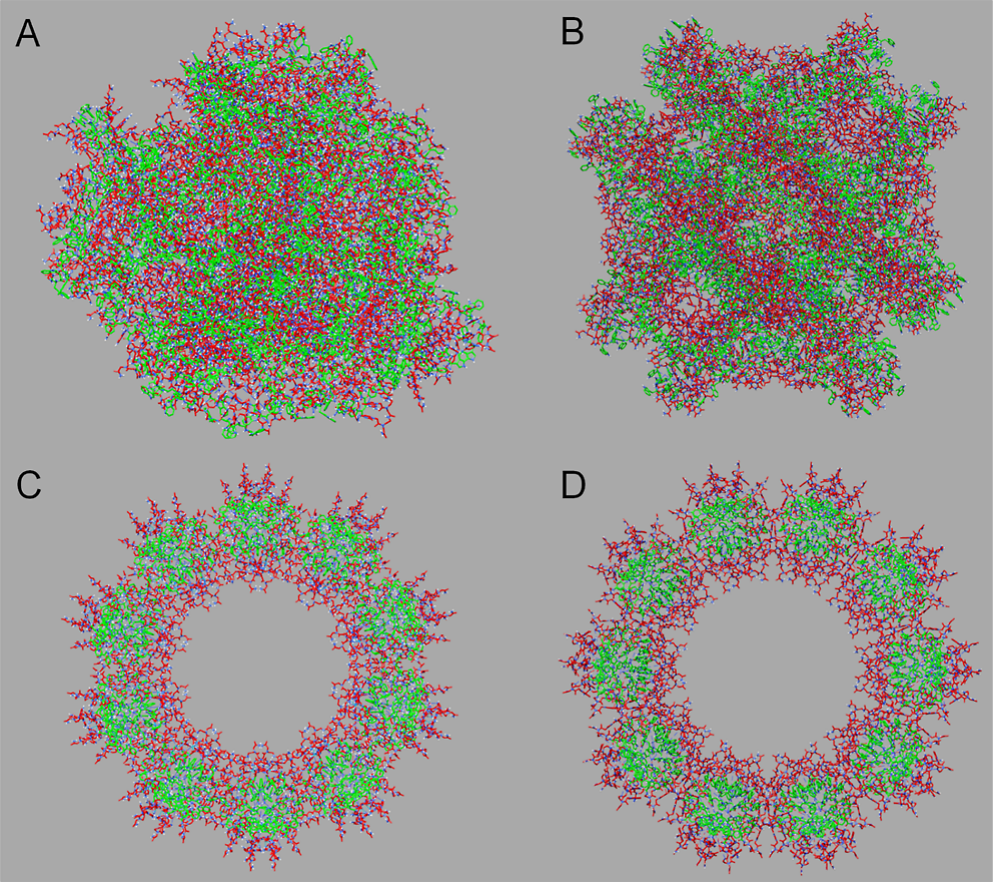
Sphere-like models of WWgc and WWat assemblies obtained with the Hsymdock webserver.^38^ Models of WWgc and WWat inhomogeneous sphere-like aggregates are shown in (A,B) and have a diameter of approximately 160 and 156 Å, respectively. Models of WWgc and WWat circular sphere-like aggregates are reported in (C,D) and possess approximately diameters 146 and 153 Å long, respectively. In the diverse panels, tryptophan residues are colored in green and PNA monomers are colored in red.

To obtain speculative models of sphere-like aggregates, 3D coordinates of WWgc and WWat monomers were employed as an input for the Hsymdock webserver^38^ (Figure 7A–D). It can be envisioned formation of diverse sphere-like aggregates that can be either stabilized by H-bonds involving both PNA polar hydrogens and Trp indolic H_N_, or spherical assemblies generated simply by H-bonds mediated by PNA bases. In the first scenario (Figure 7A,B), WWgc and WWat aggregates form inhomogeneous sphere-like complexes, in which several H-bonds between the W indolic H_N_ and the oxygen atoms of PNA backbone or Cytosine/Thymine bases are possible (Supporting Information Figure S12A,B). Instead, circular aggregates can be obtained (Figure 7C,D) if the aromatic W moieties are masked by hydrophilic “gc” or “at” bases that tend instead to stay solvent exposed. In the latter models, only intermolecular H-bonds between adjacent PNA bases, not attributable to canonical Watson-Crick base pairing, can be found (Supporting Information Figure S12C,D). Changes in the maximum of fluorescence emission at increasing concentration observed for WWgc are consistent with this hypothesis.

In spherical aggregates, stacking interactions involving W residues as well as PNA bases can be also observed.

Recently, it has been reported that the FFFFat peptide/PNA hybrid molecule is able to self-assemble into ribbon-like fibers^18^ generated by an antiparallel β-sheet organization of the FFFF peptide portion flanked by at/ta canonical base pairing and by twisting of several bilayers together.

In the present study, WWat showed that it forms sphere-like structures only. These diverse results could again be a consequence of tryptophan physico-chemical properties that allow for stronger stacking interactions and formation of more stable aggregates with respect to phenylalanine, as also reported in other studies in which the self-assembly of hybrid molecules containing WW or FF di-peptides has been investigated.^17,23,37,39^ In fact, as mentioned before, tryptophan is characterized by an indole ring that can be engaged into hydrogen bonding, and a large quadrupole that allows it to mediate strong π–π or cation−π interactions.

As concerning WWgc, this molecule, differently from WWat, can form fibers at 10 mg/mL concentration. This different behavior could be explained by considering that formation of gc/cg Watson-Crick base pairing is stabilized by 6 H-bonds and thus is energetically favorable with respect to the at/ta coupling, that is instead characterized by only 4 H-bonds.^40,41^ Thus, by increasing the concentration, the strength of gc/cg base pairing together with π–π stacking between aromatic amino-acids triggers a nucleation-polymerization process similar to that observed in peptide fibril formation and hypothesized in the formation of fibril in conjugates composed of WW and DNA.^23,42^

While formation of a helical fiber is possible for WWgc, in WWat molecules the strength of at/ta base pairing is not sufficient to produce a “nucleation core” and only sphere-like aggregates are observed.

Interestingly, fibers formed by WWgc appear highly helical and more regular than the ribbon-like structures seen in FFFFat. SinceWW dipeptides are shorter than FFFF units, they are unable to assemble in the ordered β-sheet arrangement adopted by FFFF, stabilized by several intermolecular H-bonds involving peptide backbone atoms. CD studies, in fact, do not point out the presence of a β-structuration in the WW/PNA conjugates that were here investigated.^18^

Studies on a PEG conjugated WWWW system also revealed self-assembly but, differently from what was observed for WWgc, a cross-β fibrillar organization and a backbone right-handed helical twist at increasing concentration was evidenced.^43^

Models obtained for WWgc are also consistent with fluorescence data, showing that tryptophans are more exposed to the solvent at high WWgc concentrations (i.e., in the fibers) as compared to the low concentrations, where tryptophans are embedded in a closely packed spherical aggregate.

## Conclusions

PNA-peptide conjugates can self-assemble into structures stabilized by hydrogen bonds and stacking interactions between nucleobases and amino acids. The dipeptide WW is prone to forming sphere-like structures.^23^ Conjugation to the PNA sequence gc determines a change in the morphology of the aggregates in a concentration-dependent fashion. At high concentrations, the Watson Crick hydrogen bonds between guanine and cytosine and π–π interactions between tryptophans in WWgc stabilize a nucleation core, which evolves to produce helical fibers. Results obtained suggest that in such multicomponent systems, formation of fiber occurs only when a certain concentration of monomers is reached. The fibers here reported could find application as cytotoxic compounds, similar to higher ordered structures formed by aromatic metabolites or tryptophan that exert neurotoxic action.^21^ In addition, fibers could be employed for the production of new biomaterials, such as hydrogel to be applied in tissue engineering.

## Materials and Methods

### Synthesis

The conjugates were synthesized in a 20 μM scale using solid phase Fmoc chemistry by standard protocols on the Rink amide (loading: 0,3–0,8 mmol/g) resin.^10^ To synthesize the conjugates with the PNAs at the C-terminus, we coupled to the resin the PNA monomers first and the amino-acids then. To synthesize the conjugates with the amino acids at the C-terminus, we linked the amino acids to the resin; the PNA monomers were coupled to the WW anchored to the resin. All products were purified using preparative RP-HPLC chromatography on a Jupiter 10 μm Proteo 90 Å, LC Column 100 ×21.2 mm, *E*_a_ and then analyzed using Applied Biosystems 4700 Proteomics Analyzer instrument mass spectrometer and analytical RP-HPLC chromatography on a Sepachrom Vydamas 5μ C18 100 Å 150 × 4.6 mm column. To purify the WW conjugates, a gradient of CH_3_CN (0.1% v/v TFA) in H_2_O (0.1% v/v TFA) from 10 to 50% in 30 min was used. After HPLC purification, to remove residual TFA, samples were lyophilized three times: the first to remove HPLC solvents, the second from a mixture of H_2_O/CH_3_COOH 7/3, and the third from H_2_O.

Retention time and data from the analysis of mass spectra are reported below for each product.

WWgc:

Retention time = 13.5 min

Calculated mass (Da) for [M + H]^+^ 932.4022; found: 932.6880 [M + H]^+^ and 954.6306 [M + Na]^+^

WWat:

Retention time = 14.3 min. Calculated mass (Da) for [M + H]^+^ 931.4069; found: 931.6440 [M + H]^+^ and 953.6887 [M + Na]^+^

gcWW:

Retention time = 16.5 min. Calculated mass (Da) for [M + H]^+^ 932.4022 found: 954.6400 [M + Na]^+^

atWW:

Retention time = 18 min. Calculated mass (Da) for [M + H]^+^ 931.4069; found: 953.6484 [M + Na]^+^

### UV–Visible Absorption

The UV–vis absorption measurements were conducted on a spectrophotometer Jasco V-530. UV spectra were acquired in a range of 240–350 nm; the absorbance values at 260 nm were employed to evaluate the samples concentration. Extinction coefficient (ε) values for the PNA–peptide conjugates calculated based on the values reported in the literature of each base and amino acid at 260 nm are 26591.2 for WWgc and gcWW and 31131.2 for WWat and atWW M^–1^ cm^–1^. The concentration was calculated applying the Lambert–Beer law.

### Circular Dichroism

The secondary structure of assembled WWgc and WWat samples was determined by CD spectroscopy. The CD spectra were recorded using a Jasco J-815 spectropolarimeter (Jasco, Easton, MD) at 25 °C from 190 to 320 nm (1 nm bandwidth and 0.1 nm resolution). The measurements were executed in a 0.1 mm optical path quartz capillary cuvette at 25 °C in ammonium sulfate buffer pH 5.5 or in water at a concentration 10× CAC. CD spectra are reported in molar ellipticity and measured in units of mdeg as a function of wavelength.

### Fluorescence

Solutions for fluorescence measurements were obtained dissolving samples in water at a final concentration of 10 mg mL^–1^. The experiments were conducted on a spectrofluorometer Fluorolog Jobin Yvon Horiba using 1 cm path length. The emission spectra were registered exciting at different wavelengths in a range between 300 and 390 nm; the excitation spectra were acquired at different emission wavelengths from 410 to 436 nm. Excitation and emission spectra were acquired using the same slits parameters; each product needed different slits. The data plotting was performed by OriginLab software.

### Atomic Force Microscopy

Samples were dissolved in H_2_O or in ammonium sulfate buffer (at 4 and 10 mg mL^–1^) and in the presence or absence of 10% of MeOH. Then, 50 μL was spotted onto a freshly cleaved Muscovite mica disk and incubated for 5 min at room temperature. The disk was washed with H_2_O, and it was finally dried under a gentle nitrogen stream in 5 min.

Muscovite mica disk containing the samples was placed onto a Multimode AFM with a NanoScope V system (Veeco/Digital Instruments) operating in Tapping Mode using standard antimony(n)-doped Si probes (*T*: 3.5–4.5 mm, *L*: 115.135 mm, *W*: 30–40 mm, *f*_0_: 313–370 kHz, *k*: 20–80 N/m) (Bruker). Samples were analyzed with the scanning Probe Image Processor [SPIP Version 5.1.6 (released April 13, 2011)] data analysis package. SPIP software was also employed to analyze the chirality of the fibers.

### NMR Spectroscopy

NMR experiments were recorded on a Bruker ADVANCE 500 MHz spectrometer equipped with a cryoprobe at 298 K for WWgc samples at concentrations equal to 10 and 4 mg/mL in 600 μL H_2_O/D_2_O (deuterium oxide, 98% D, Sigma-Aldrich, Milan-Italy) (90/10 *v*/*v*). The pH value of each sample was adjusted to 5 with a dropwise addition of NaOH. The following NMR spectra were acquired: 1D [^1^H], 2D [^1^H, ^1^H] TOCSY (Total Correlation Spectroscopy) with a mixing of 70 ms,^44^ and 2D [^1^H, ^1^H] NOESY (Nuclear Overhauser Enhancement Spectroscopy)^45^ with a mixing time of 300 ms. NMR spectra were recorded with 32–64 scans, 256 FIDs in *t*1, 2048 data points in *t*2. Water suppression was obtained with Excitation Sculpting.^46^ Chemical shifts were referenced to the water peak at 4.70 ppm. Spectra were processed with Topspin 4.2 (Bruker, Milan, Italy) and analyzed with NEASY contained in CARA.^47^

### Computational Studies

The WW peptide was assembled with the “build structure” tool of UCSF Chimera (version 1.16)^33^ by imposing backbone dihedral angles canonical of an extended structural organization (ϕ = −139°, ψ = 135°). This di-peptide was used as input receptor and ligand in Haddock (Haddock 2.2 webserver)^34^ to generate atomic coordinates of a dimer. During docking calculations, the peptide N-terminus was considered positively charged and the Trp residues were set as active. The first step of docking protocol included a rigid body energy minimization that provided 1000 output structures. In the following step, a semi-flexible simulated annealing of the best 200 solutions and a final refinement in water were carried out. Finally, the 200 models were subjected to a clusterization procedure with an rmsd cut-off value of 5 Å.^34^ The best structure of the best cluster (i.e., number 1 from cluster 4 with Haddock score = −63.95) was employed to form WWgc and WWat units.

The atomic coordinates for the PNA monomers were extracted from the pdb entry 2KVJ, representing the structure of a right-handed backbone γ-methylated PNA duplex.^35^ Next, the γ methyl groups were deleted from the PNA skeleton and the backbone of resulting structures was minimized by means of the Molecular Modelling Toolkit minimization module of UCSF Chimera^48^ by performing 1000 steepest descent cycles and 100 conjugate gradients cycles (step size 0.02 Å).

To generate fibers, in the third step, the gc/cg duplex was linked by peptide bonds to the WW model obtained with Haddock: a bond between the backbone NH amide group of guanine and the W2 CO groups in each monomeric WW unit was edited in Chimera with default parameters (C–N length 1.33 Å, ω angle equal to 180°).

In detail, 19 monomeric units were combined together to form a fiber. The resulting structure was subjected to a further energy minimization step by Chimera (1000 steepest descent cycles and 100 conjugate gradients cycles) by keeping fixed Guanine and Cytosine atoms to avoid disruption of original base-pairing geometry.

To generate models of sphere-like aggregates, 3D coordinates of a monomeric WW dipeptide were conjugated C-terminally to “gc” or “at” PNA dimers extracted from the structure with PDB code 2KVJ.^35^ Sphere-like assemblies were built by the Hsymdock web server (http://huanglab.phys.hust.edu.cn/hsymdock/)^38^ that is based on a hierarchical fast Fourier transform symmetric docking algorithm^49^ and an iterative distance-dependent scoring function for protein–protein interactions.^50^ Hsymdock calculates models of molecular aggregates with a Cn (circular) or Dn (dihedral) symmetry; oligomeric structures assembled with a Cn symmetry are characterized by the rotation around a single axis of one subunit; instead, oligomeric structures with a Dn symmetry are characterized by a two-fold symmetry in which a Cn axis is combined with a perpendicular axis.

To consider the contribution of different networks of intermolecular interactions and to obtain large aggregates, we adopted two different protocols. According to the first strategy, WWgc or WWat monomers were used as input for a *C*2 symmetry (circular symmetry docking assembly of two units) run, and the best calculated Cn oligomer (according to the docking score) was next used as input for a further identical run (leading to an aggregate made up of four units). This protocol was repeated for a total of nine runs until sphere-like aggregates including 512 WWgc or WWat units were assembled. In the second protocol, WWgc or WWat units were first used as input for a D10 symmetry run (docking by dihedral symmetry combining 20 units together), and then one of the resulting best solutions was employed as input for a further C10 circular symmetry calculation (final number of assembled units equal to 200).^38^

## Supporting Information Available

HPLC profiles and mass spectra of pure compounds, fluorescence spectra of atWW and gcWW, tapping mode AFM analyses, diameter and length analysis of WWgc samples, NMR spectra of WWgc at different concentrations, and details of WWgc and WWat sphere-like models (PDF)
