# Supplementary material for: Tryptophan-PNA gc Conjugates Self-Assemble to Form Fibers
Source: Bioconjug Chem. 2023 Jul 24;34(8):1429–38. doi: 10.1021/acs.bioconjchem.3c00200 (PMC10436247; doi:10.1021/acs.bioconjchem.3c00200)
Supplement: Supplementary file 1 — bc3c00200_si_001.pdf [file bc3c00200_si_001.pdf]

Supporting information for

Tryptophan-PNA gc conjugates self-assemble to form fibers

Andrea Mosseri[a], Maria Sancho-Albero[b], Flavia Anna Mercurio[c], Marilisa Leone[c], Luisa De Cola[a,b], Alessandra Romanelli[a]*

[a] Dipartimento di Scienze Farmaceutiche, Università degli Studi di Milano, via Venezian 21, 20133 Milano, Italy.

[b] Department of Molecular Biochemistry and Pharmacology, Istituto di Ricerche Farmacologiche Mario Negri IRCCS, 20156, Milano, Italy.

[c] Istituto di Biostrutture e Bioimmagini – CNR, via Mezzocannone 16, 80134 Naples, Italy.

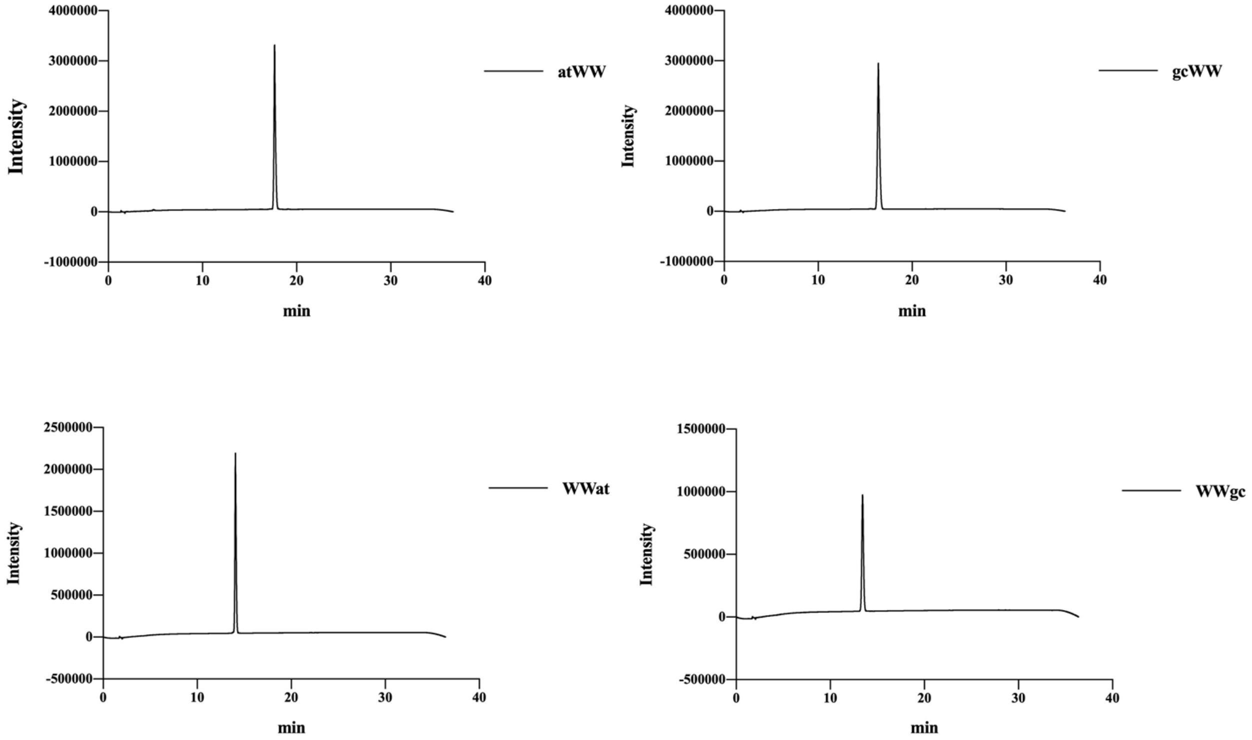

Figure S1. HPLC profiles of pure compounds. Column: Sepachrom Vydamas 5µ C18 100 Å 150x4.6 mm; method: gradient of CH_3CN (0.1% v/v TFA) in H_2O (0.1% v/v TFA) from 10% to 50% in 30 minutes.

Figure S2. MALDI-TOF spectra of PNA-peptide conjugates

gcWW

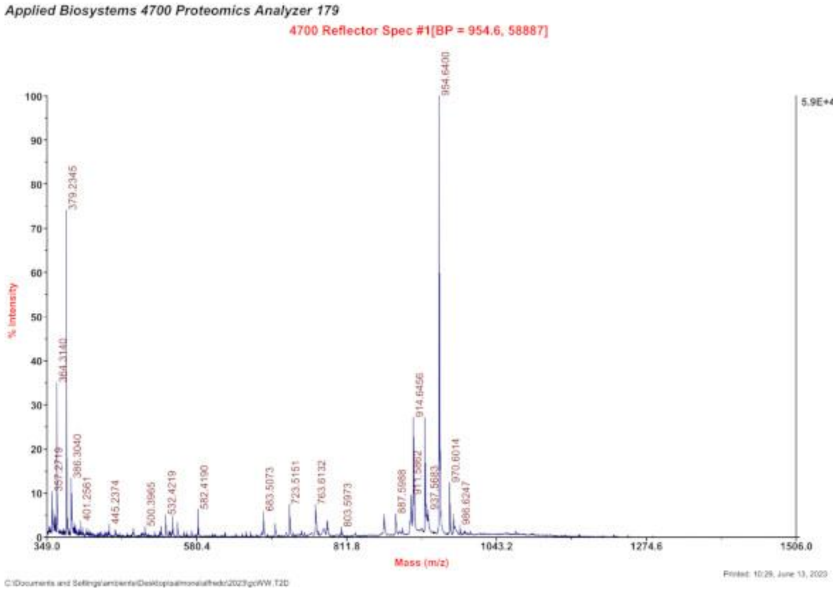

atWW

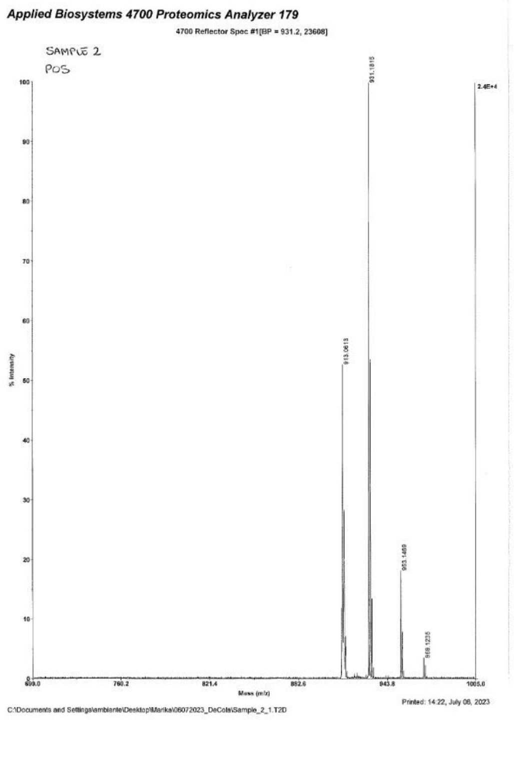

WWat

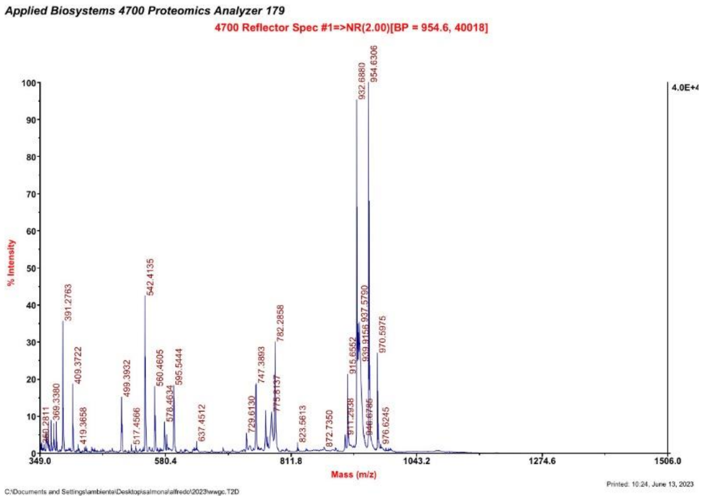
Applied Biosystems 4700 Proteomics Analyzer 179
4700 Reflector Spec #1=>NR(2.00)[BP = 954.6, 40018]
4.0E+4
% Intensity
Mass (m/z)
C:\Documents and Settings\ambiente\Desktop\salmona\alfredo\2023\wwgc.T2D
Printed: 10:24, June 13, 2023

WWgc

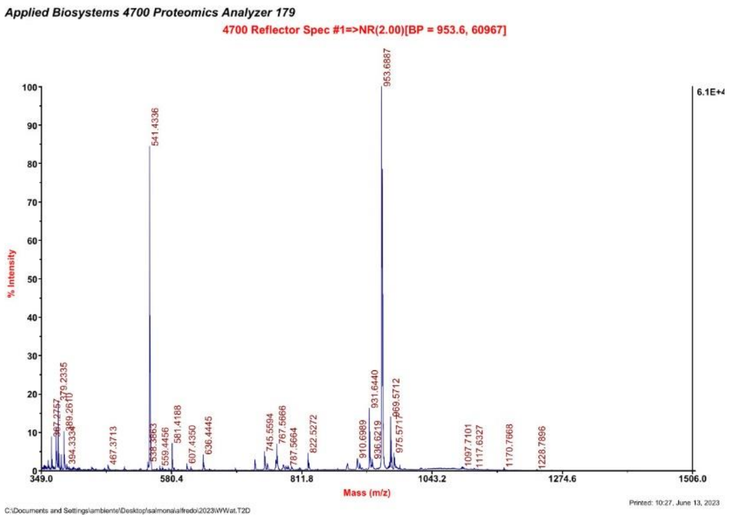
Applied Biosystems 4700 Proteomics Analyzer 179
4700 Reflector Spec #1=>NR(2.00)[BP = 953.6, 60967]
6.1E+4
% Intensity
Mass (m/z)
C:\Documents and Settings\ambiente\Desktop\salmona\alfredo\2023\WWat.T2D
Printed: 10:27, June 13, 2023

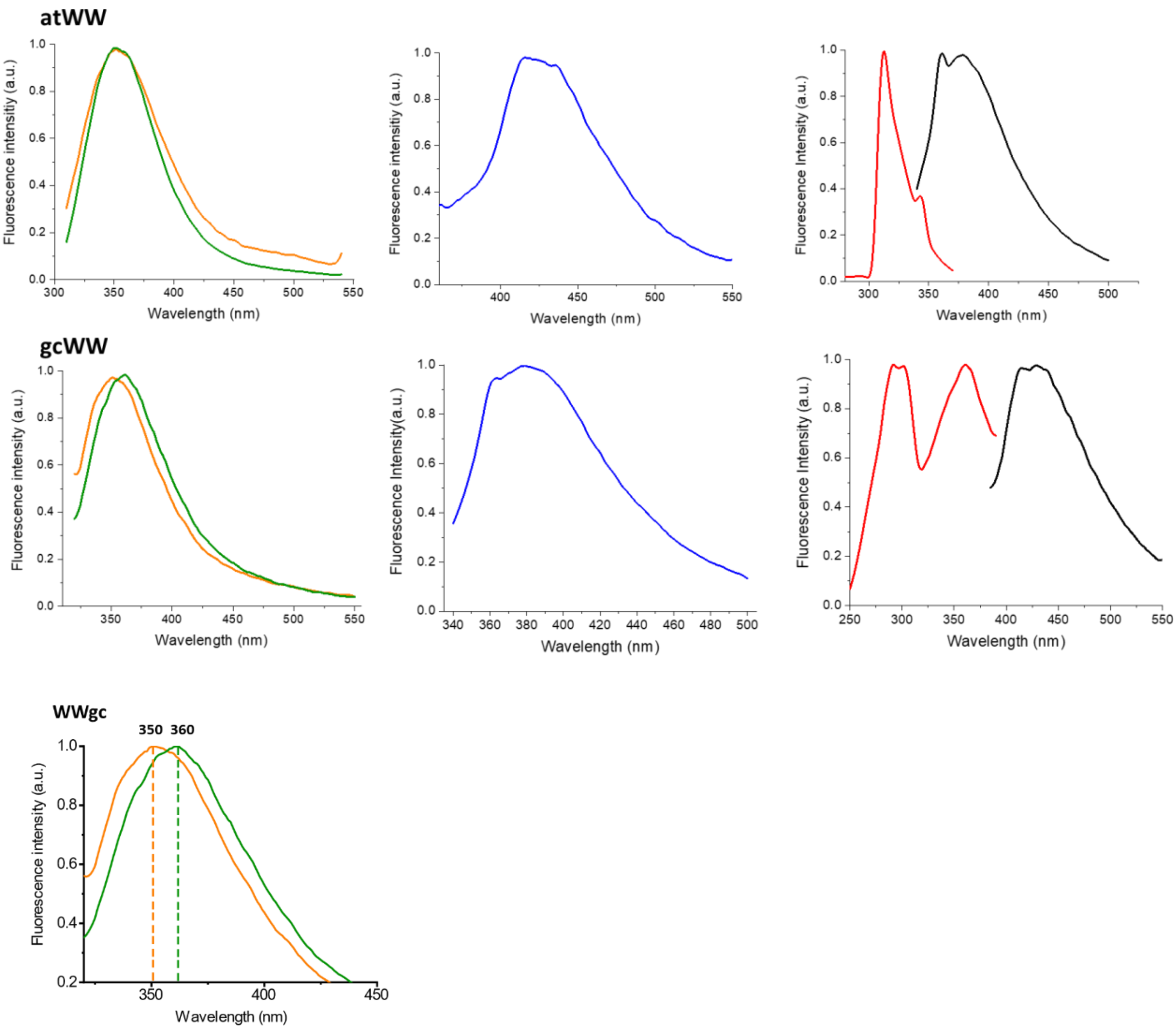

Figure S3. Fluorescence spectra of atWW and gcWW in water (top and middle). From left to right: emission spectrum λ_{ex}: 280 nm (green line: 10 mg/mL; orange line: 1 mg/mL), emission spectrum λ_{ex}: 330 nm (blue line) at 10 mg/mL, excitation (red line) and emission (black line) spectra with λ_{ex}: 360 nm and λ_{em}: 450 nm at 10 mg/mL The bottom panel is a zoom of the emission spectra of WWgc reported in Figure 3, highlighting the 10 nm shift in the emission spectrum at increasing concentration.

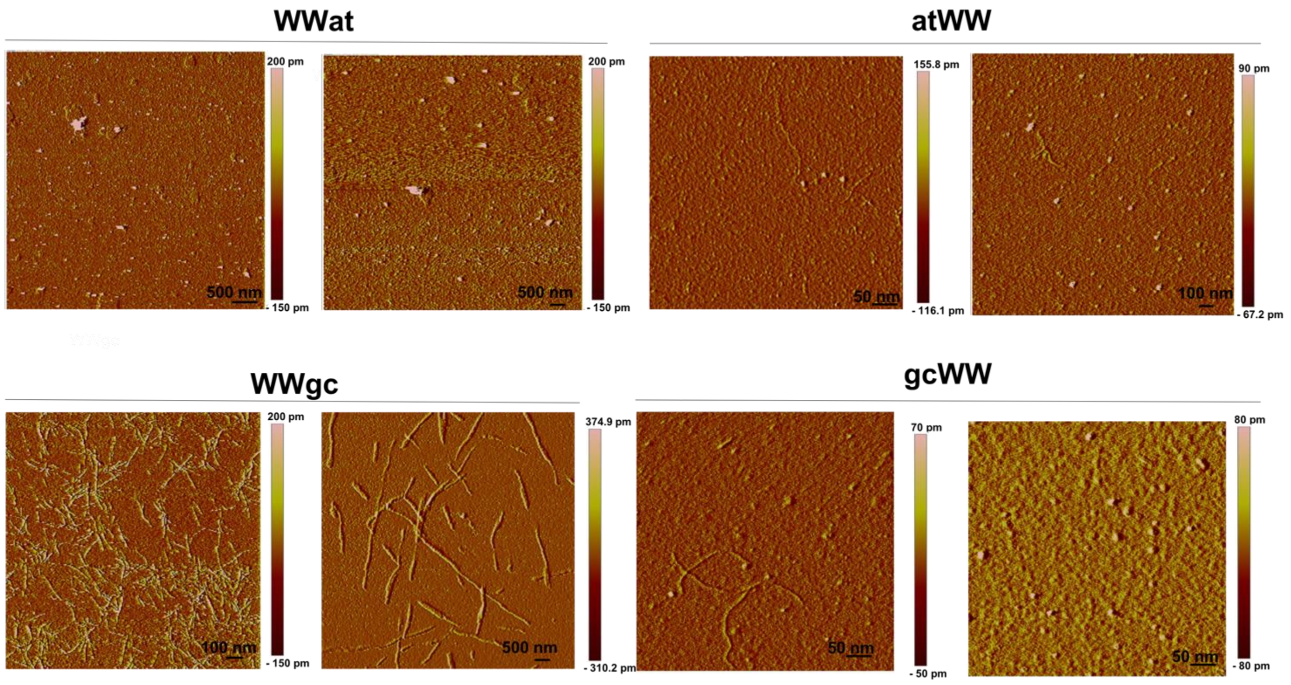

Figure S4. Tapping mode AFM analysis of WWat, atWW, WWgc, gcWW aggregates in water (10 mg/mL).

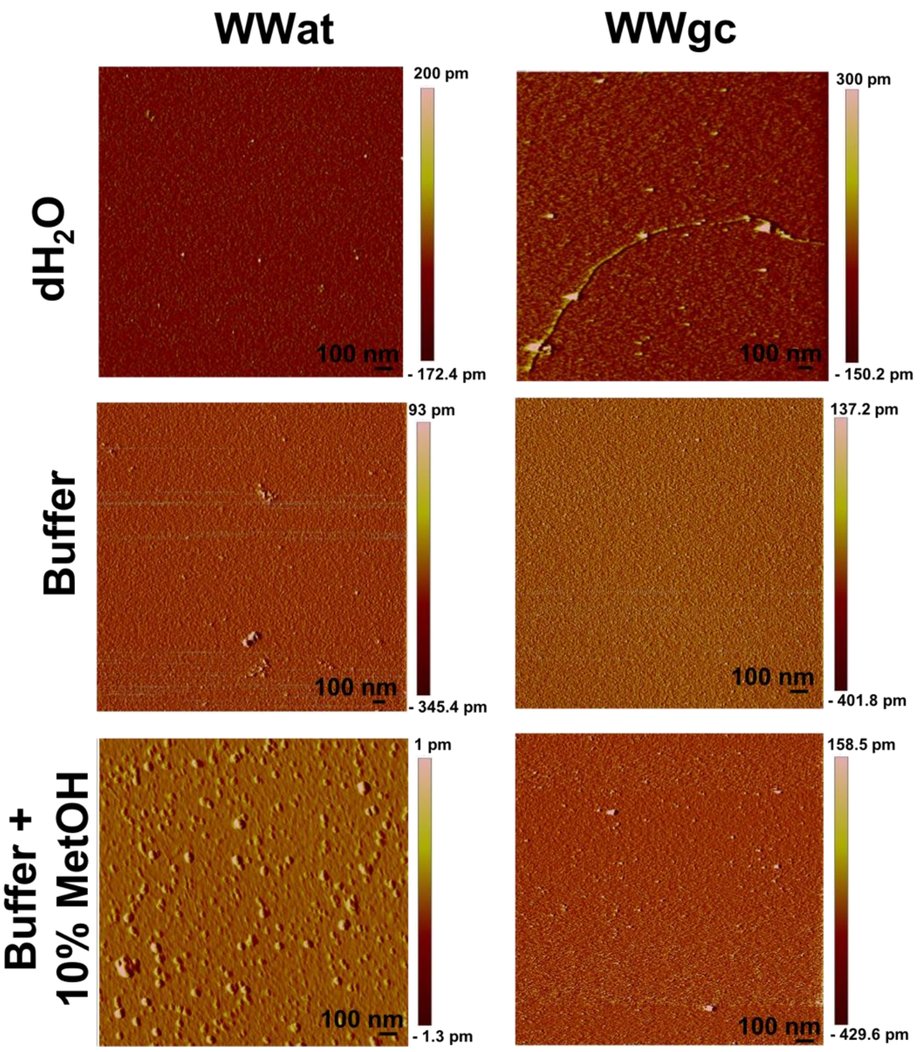

Figure S5. Tapping mode AFM analysis of WWat, and, WWgc aggregates at 4 mg/mL in water, buffer (pH 5.5) and buffer (pH 5.5) with MeOH 10 %.

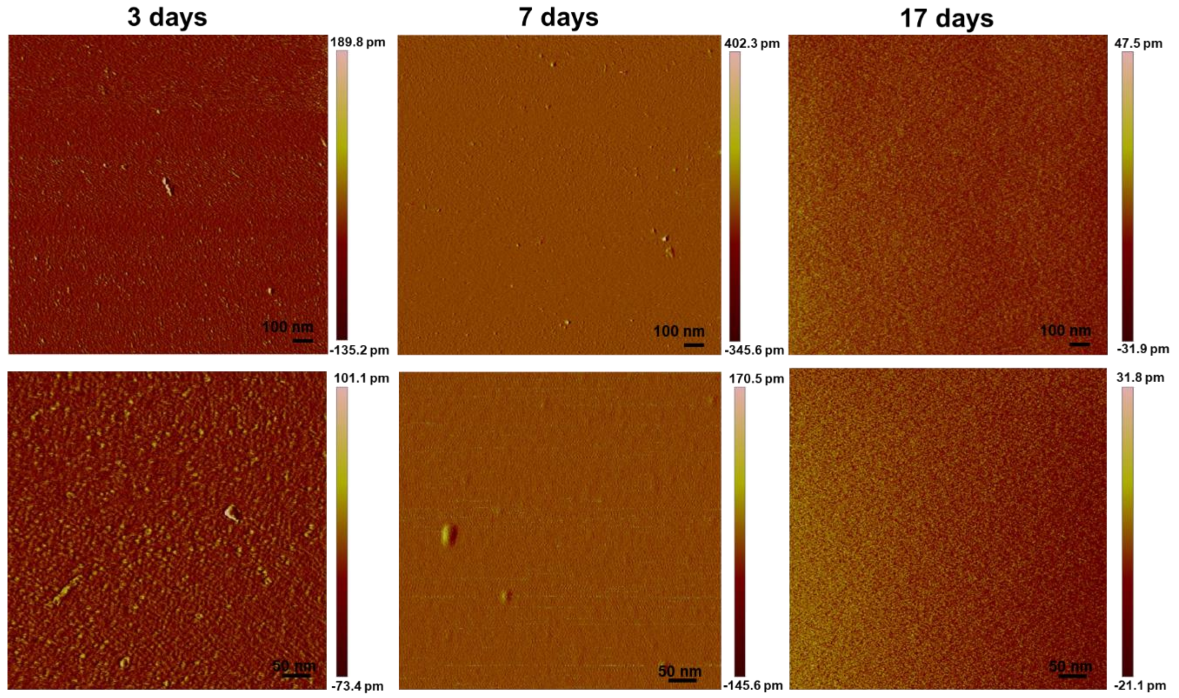

Figure S6. Tapping mode AFM analysis of WWgc aggregates at 4 mg/mL incubated in water for different times.

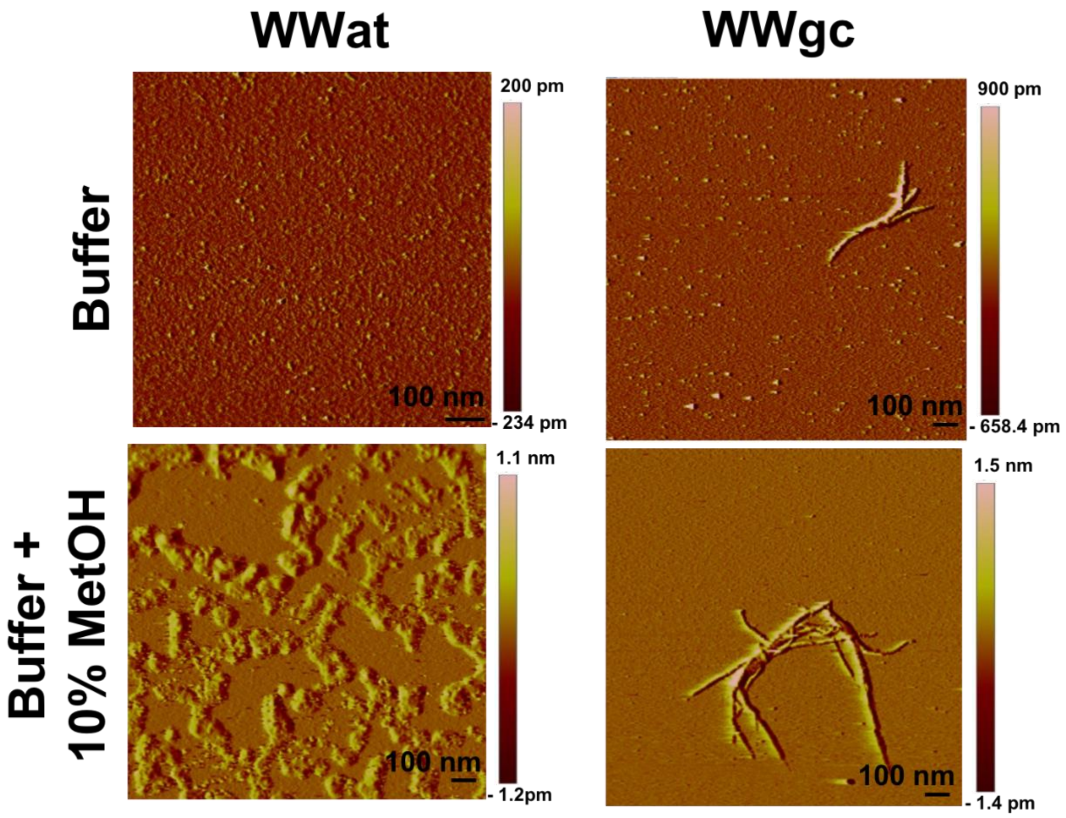

Figure S7. Tapping mode AFM analysis of WWat, and, WWgc aggregates at 10 mg/mL in buffer and buffer with MeOH 10 %.

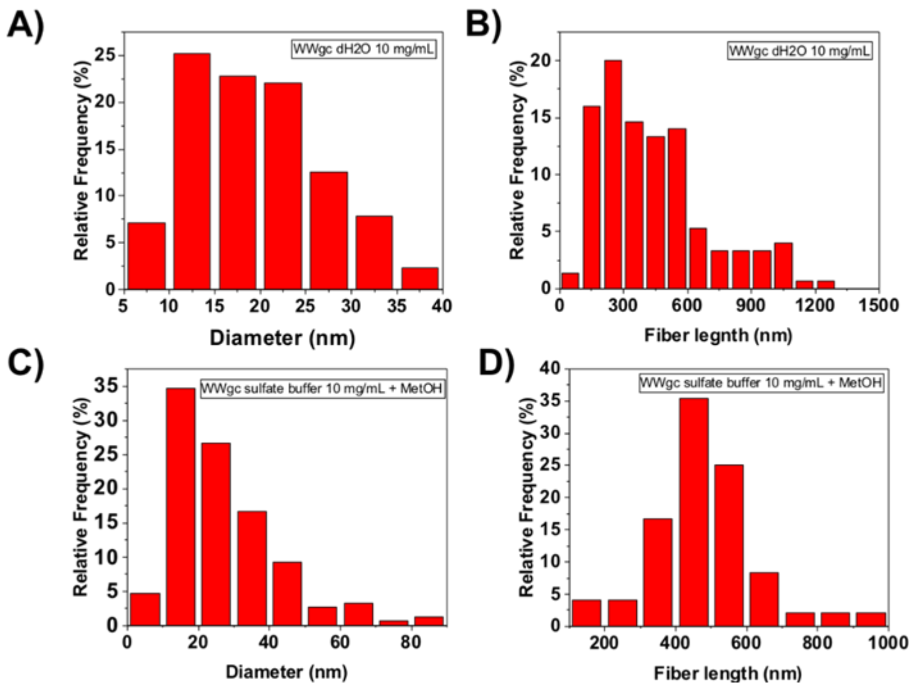

Figure S8. Diameter and fiber length analysis of WWgc samples when they were dissolved in water or in buffer with 10 % methanol.

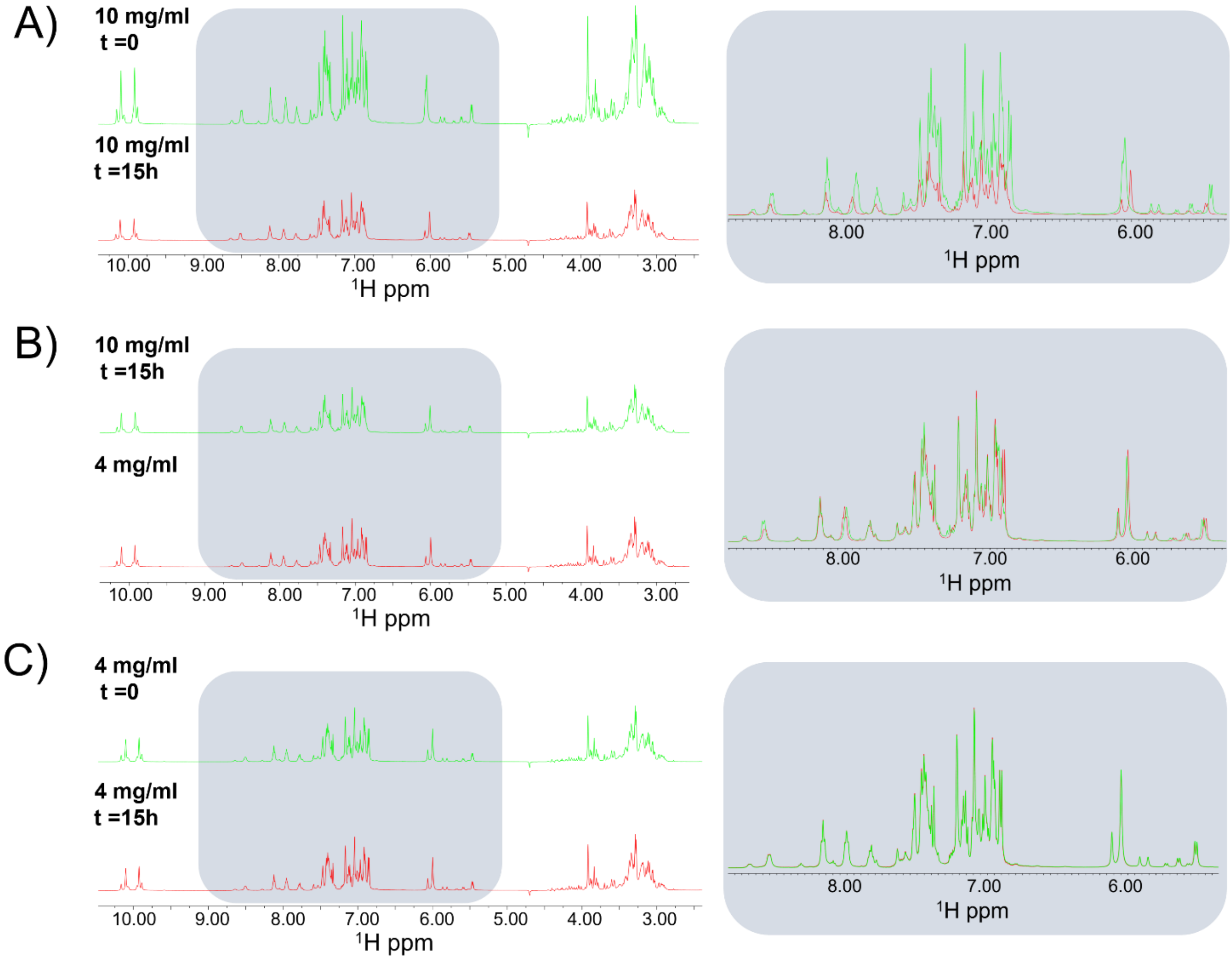

Figure S9. Comparison of 1D [^{1}H] NMR spectra of (A) WWgc (10 mg/mL) at different incubation times, (B) of WWgc (10 mg/mL) after 15h incubation and WWgc (4 mg/mL) and (C) of WWgc (4 mg/mL) at different incubation times. The spectral regions evidenced by light blue rectangles in each panel are shown as superpositions in the right inserts.

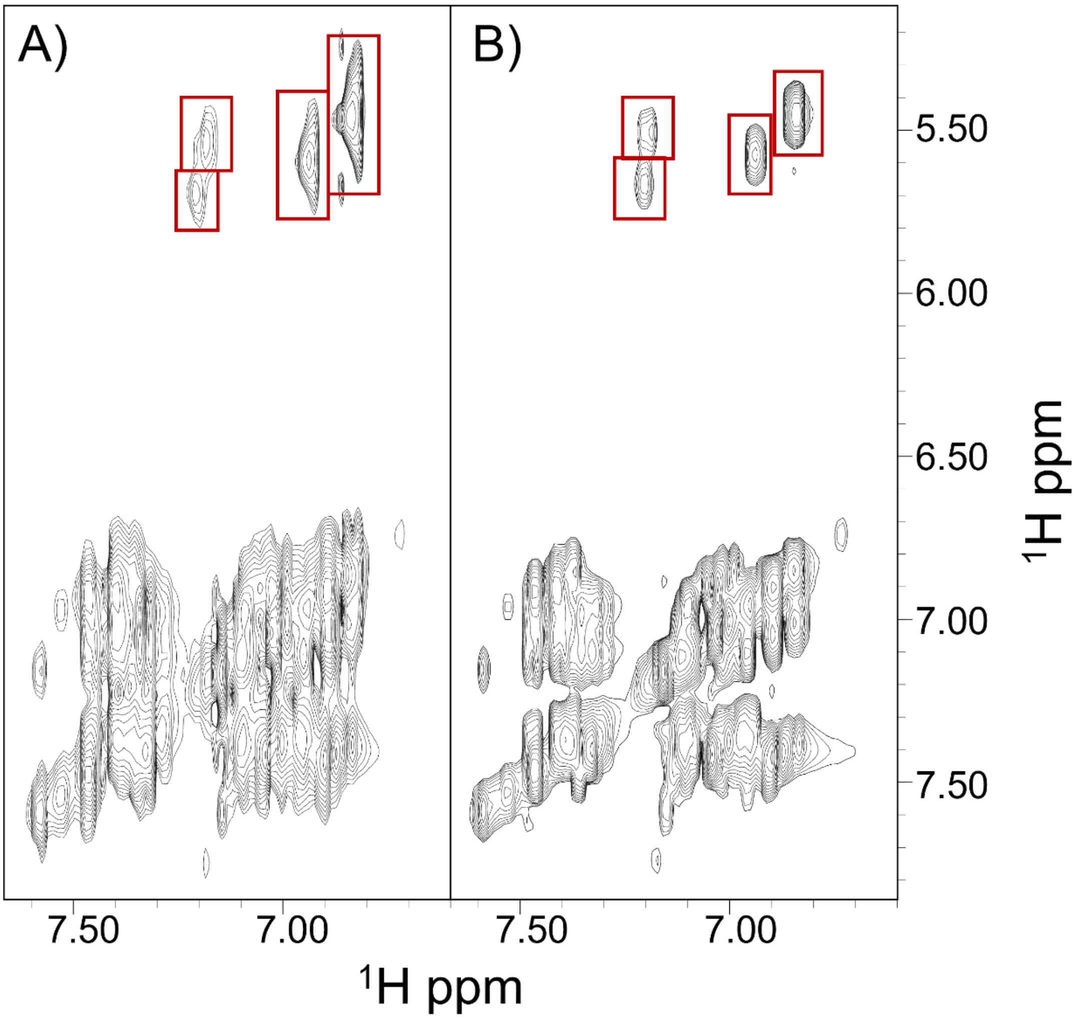

Figure S10. 2D [^{1}H-^{1}H] TOCSY spectra of WWgc at concentrations equal to 10 mg/mL (A) and 4 mg/mL (B). Correlations in between aromatic H5 and H6 cytosine protons in the four rotamers are highlighted with red boxes.

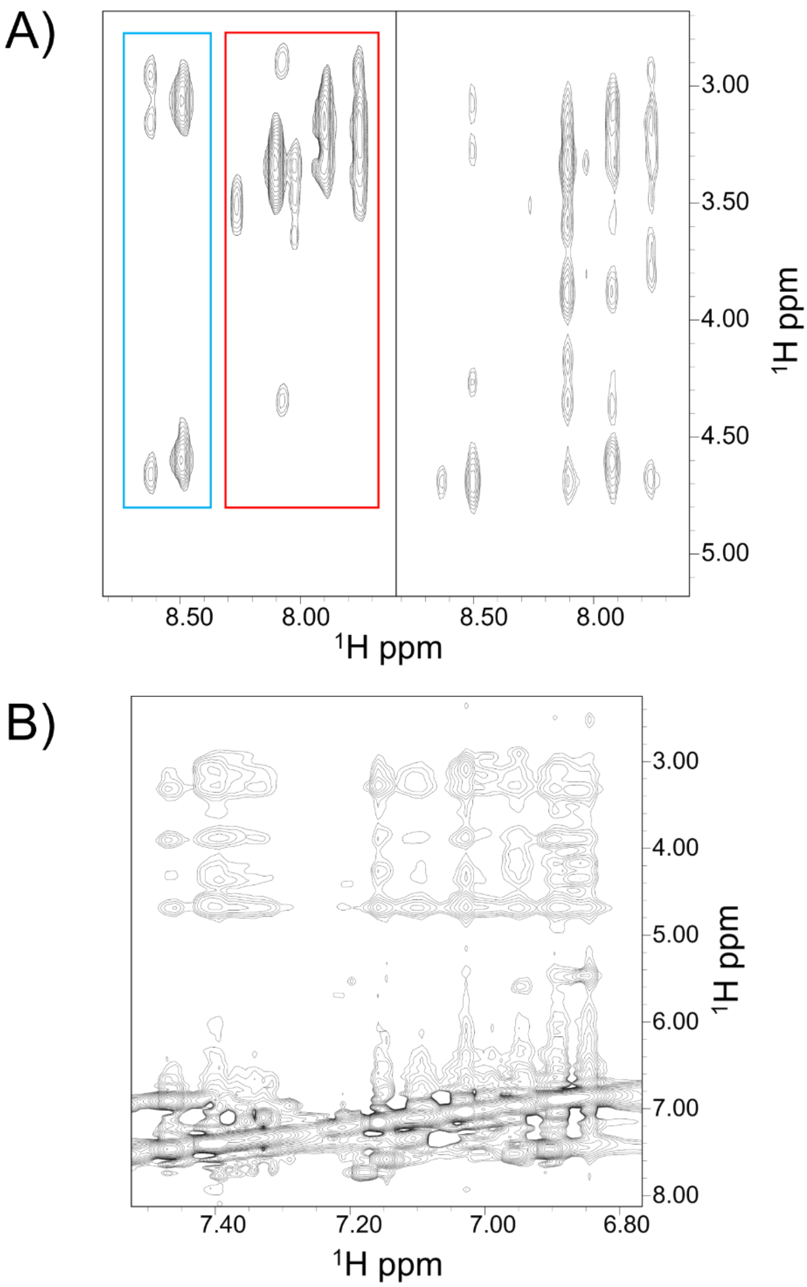

Figure S11. (A) Comparison of HN-high field correlation regions of TOCSY (left) and NOESY (right) spectra of WWgc (10 mg/mL). Blue and red rectangles in the TOCSY spectrum indicate Tryptophans and PNA residues spin systems, respectively. (B) Aromatic region of the NOESY spectrum of WWgc (10 mg/mL).

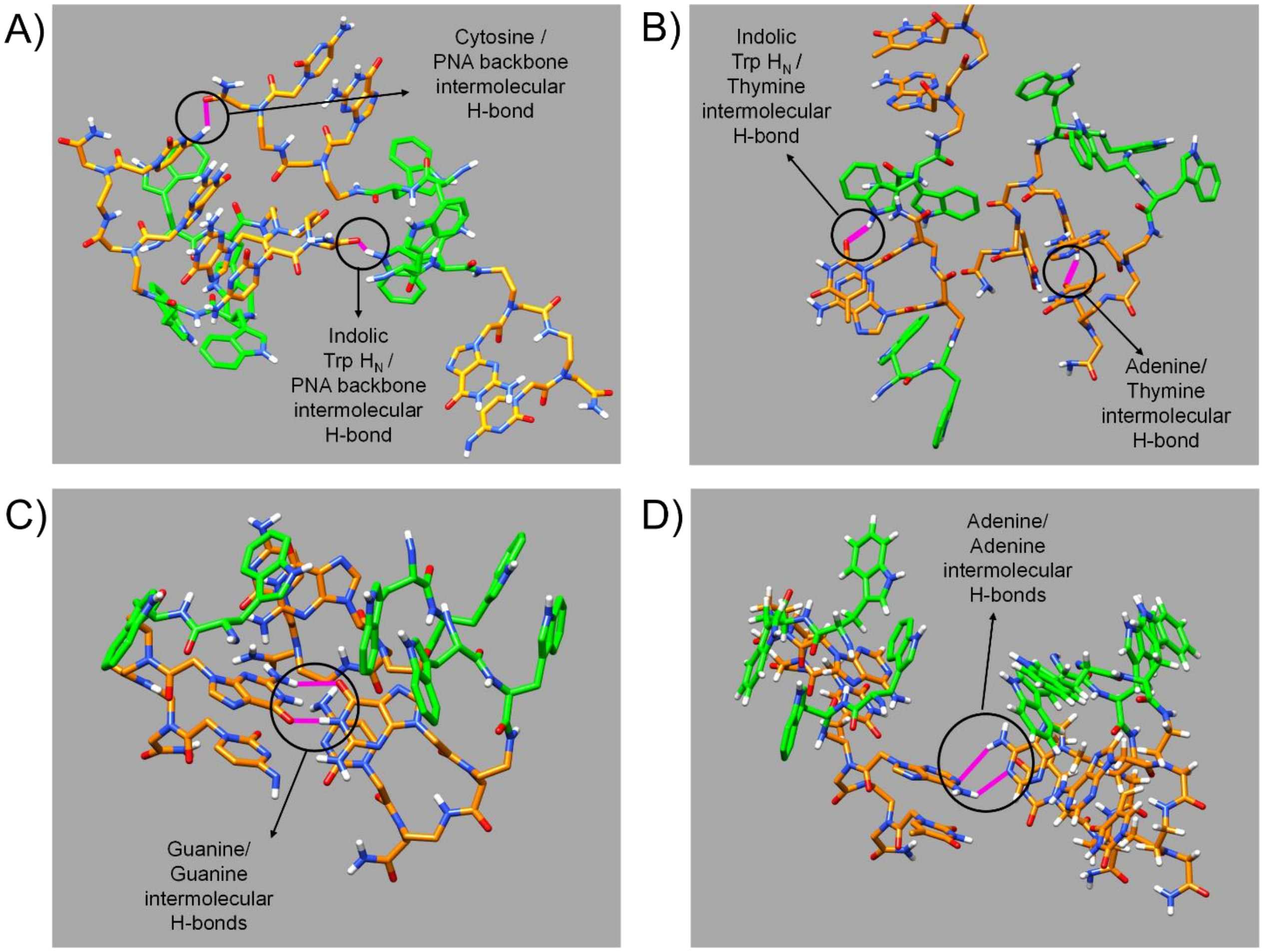

Figure S12. Details of WWgc and WWat sphere-like models reported in figure 7A-D of the main text in which different types of intermolecular H-bonds are highlighted. More precisely, panel (A) is related to the WWgc inhomogeneous sphere-like model shown in figure 7A; panel (B) is related to WWat inhomogeneous sphere-like model shown in figure 7B; panel (C) is related to WWgc circular sphere-like model shown in figure 7C; panel (D) is related to WWat circular sphere-like model shown in figure 7D. In the diverse panels only a few units composing the whole models reported in Figure 7A-D are shown to better visualize H-bonds. Trp and PNA residues are colored in green and orange, respectively, and H-bonds are displayed in magenta.
